# Pindolol Rescues Anxiety-Like Behavior and Neurogenic Maladaptations of Long-Term Binge Alcohol Intake in Mice

**DOI:** 10.3389/fnbeh.2019.00264

**Published:** 2019-11-29

**Authors:** Omkar L. Patkar, Arnauld Belmer, Kate Beecher, Angela Jacques, Selena E. Bartlett

**Affiliations:** ^1^Addiction and Obesity Laboratory, Department of Clinical Sciences, Queensland University of Technology, Brisbane, QLD, Australia; ^2^Institute of Health and Biomedical Innovation (IHBI), Queensland University of Technology, Brisbane, QLD, Australia

**Keywords:** pindolol, serotonin, 5-HT1A/1B receptor, neurogenesis, long-term alcohol intake, anxiety-like behavior

## Abstract

Long-term binge alcohol consumption alters the signaling of numerous neurotransmitters in the brain including noradrenaline (NE) and serotonin (5-HT). Alterations in the signaling of these neuronal pathways result in dysfunctional emotional states like anxiety and depression which are typically seen during alcohol withdrawal. Interestingly, studies have demonstrated that the development of alcohol-induced negative affective states is linked to disrupted neurogenesis in the dentate gyrus (DG) region of the hippocampus in alcohol-dependent animals. We have previously shown that modulation of NE and 5-HT activity by pharmacological targeting of β-adrenoreceptors (β-ARs) and 5-HT1A/1B receptors with pindolol reduces consumption in long-term alcohol-consuming mice. Since these receptors are also involved in emotional homeostasis and hippocampal neurogenesis, we investigated the effects of pindolol administration on emotional and neurogenic deficits in mice consuming long-term alcohol (18 weeks). We report that acute administration of pindolol (32 mg/kg) reduces anxiety-like behavior in mice at 24 h withdrawal in the marble-burying test (MBT) and the elevated plus-maze (EPM). We also show that chronic (2 weeks) pindolol treatment (32 mg/kg/day) attenuates alcohol-induced impairments in the density of immature neurons (DCX^+^) but not newborn cells (BrdU^+^) in the hippocampal DG. Pindolol treatment also restores the normal proportion of newborn proliferating cells (BrdU^+^/Ki67^+^/DCX^−^), newborn proliferating immature neurons (BrdU^+^/Ki67^+^/DCX^+^) and newborn non-proliferating immature neurons (BrdU^+^/Ki67^−^/DCX^+^) following long-term alcohol intake. These results suggest that pindolol, through its unique pharmacology may rescue some but not all deficits of long-term alcohol abuse on the brain, adding further value to its properties as a strong pharmaceutical option for alcohol use disorders (AUDs).

## Introduction

Alcohol abuse is highly prevalent in society with significantly higher rates of co-occurrence with mood disorders including depression and anxiety (Penick et al., 1988; Ross, 1995; Hall et al., 1999). Increased alcohol intake has been observed in animal models of anxiety and depression (Huot et al., 2001; Rezvani et al., 2002) with basal anxiety levels being key predictors in the development of alcohol dependence. In addition to altered affective states, recent studies have also demonstrated deficits in hippocampal neurogenesis in the dentate gyrus (DG) in animal models of emotional disorders (Mineur et al., 2007; Petrik et al., 2012) and alcohol consumption (Nixon and Crews, 2004; He et al., 2005), suggesting that maintenance of normal affective states and hippocampal neurogenesis may be governed by common neurochemical mechanisms.

There is an abundance of literature suggesting that dysregulation in noradrenaline (NE) and serotonin (5-HT) signaling in the brain contributes to altered affective states including anxiety and depression (Gorelick, 1989; Gallant, 1993; George, 1994; Gilpin and Koob, 2010; Petrakis et al., 2012; Silberman et al., 2012). Moreover, studies with animal models of alcohol intake and human alcoholics also implicate dysfunctional NE (Smith and Aston-Jones, 2008; Gilpin and Koob, 2010; Becker, 2012) and 5-HT (Gallant, 1993; Lê et al., 2002; Commons et al., 2003) signaling in the development of negative affective states during withdrawal. As a corollary, pharmacological blockade of altered NE and 5-HT function not only remediated withdrawal-induced emotional dysregulation, but also facilitated a reduction in alcohol consumption (Walker et al., 2008; Gilpin and Koob, 2010; Patkar et al., 2017; Belmer et al., 2018).

Treatment of altered emotional states involves the rescue of abnormally low 5-HT and NE levels mostly by blocking their reuptake in the presynaptic neuron with the use of either selective NE reuptake inhibitors (SNRIs) or selective 5-HT reuptake inhibitors (SSRIs; Naranjo and Knoke, 2001; Baldwin, 2006; Ossewaarde et al., 2011). Also, the use of selective NE (Silberman et al., 2012; Butler et al., 2014) and 5-HT receptor (Montgomery et al., 1991; Belmer et al., 2018) agonist and antagonists have been reported to enhance monoamine neurotransmission. Increasing the concentrations of 5-HT and NE not only alleviates symptoms of anxiety and depression (Silberman et al., 2012; Nautiyal et al., 2016; Belmer et al., 2018), but also markedly enhances adult neurogenesis in the DG region of the hippocampus (Santarelli et al., 2003; Jhaveri et al., 2010; Masuda et al., 2012; Belmer et al., 2018). Some studies even argue that the efficacy of antidepressants is thought to be mediated by enhancements in neurogenesis (Santarelli et al., 2003).

A huge body of evidence has implicated the role of 5-HT1 receptors particularly 5-HT1A in the modulation of 5-HT activity in response to SSRI treatment that contributes to improvements in altered emotional and neurogenic states (Diaz et al., 2013; Samuels et al., 2015). This is further supported by studies with 5-HT1A receptor agonists and antagonists that increase or decrease hippocampal neurogenesis respectively in rodents (Radley and Jacobs, 2002; Banasr et al., 2004; Samuels et al., 2015; Belmer et al., 2018). Although not as widely researched as the 5-HT1A receptor, the 5-HT1B receptor has also been shown to play a key role in aggressive (Olivier and van Oorschot, 2005) and emotional behavior as demonstrated by studies in rodents that displayed symptoms of heightened stress-induced anxiety or reduced depressive behaviors with elevation or depletion of 5-HT1B receptor expression, respectively (Nautiyal et al., 2016). Furthermore, emerging reports also suggest a role of 5-HT1B receptors in SSRI-mediated 5-HT enhancements (Nautiyal et al., 2016) and facilitation of neurogenesis (Banasr et al., 2004). Likewise increases in the levels of NE due to the action of NE antidepressants may directly increase neurogenesis through an increase in the number of neural precursor cells in the hippocampal DG (Masuda et al., 2012). This effect of NE on neurogenesis is thought to be mediated by β3-ARs which contribute to NE’s effects on neural stem cell activation and increased neurogenesis (Jhaveri et al., 2010).

Since studies robustly implicate 5-HT1 and β-adrenergic receptors in emotional regulation and hippocampal neurogenesis, coupled with other studies that show changes in these receptors with long-term alcohol intake and withdrawal (Nevo et al., 1995; Gilpin and Koob, 2010; Sari, 2013; Patkar et al., 2017; Belmer et al., 2018), we investigated the effects of pindolol, a drug with a dual mechanism of action of 5-HT1A/1B receptors and β_1_/β_2_ adrenoreceptors on anxiety-like behavior and neurogenic deficits in mice. Using the well-established drinking-in-dark paradigm (DID) in mice which facilitates high alcohol intake, symptoms of behavioral intoxication and dependence and high blood alcohol levels (Thiele and Navarro, 2014), we observed anxiety-like behavior at 24 h post-withdrawal in mice consuming long-term alcohol (12–13 weeks) on the marble-burying test (MBT) and elevated plus-maze (EPM). We further demonstrated that pindolol (32 mg/kg, i.p.) significantly reduces withdrawal-induced anxiety-like behavior to a similar level as seen in the alcohol naïve controls. We also observed that long-term alcohol intake produces significant deficits in neurogenesis as seen with a reduction in BrdU^+^ newborn cells and DCX^+^ immature neurons as compared to alcohol naive controls. We report that a 2-week chronic treatment with pindolol (32 mg/kg/day, i.p.), improves neurogenesis by increasing the number of immature neurons and restores normal proportion of newborn proliferating cells (BrdU^+^/Ki67^+^/DCX^−^), newborn proliferating immature neurons (BrdU^+^/Ki67^+^/DCX^+^) and newborn non-proliferating immature neurons (BrdU^+^/Ki67^−^/DCX^+^) in the hippocampal DG of long-term alcohol-consuming mice.

## Materials and Methods

### Animals and Housing

All procedures involving animals were pre-approved by the Queensland University of Technology animal ethics committee and the University of Queensland animal ethics committee. Five-week-old male C57BL/6J mice [Animal Resources Centre (ARC), anning Vale, WA, Australia] were individually housed under reverse light cycle conditions (lights off at 9:00 am) in a climate-controlled room. They had *ad libitum* access to food and water. Following a 2-week habituation to the housing conditions, the mice (6 week-old) were presented with alcohol during the drinking sessions.

### Drugs and Chemicals

Pindolol [1-(1H-Indol-4-yloxy)-3-(isopropylamino)-2-propanol,1-(1H-Indol-4-yloxy)-3-[(1-methylethyl)amino]-2-propanol, Sigma-Aldrich, NSW, Australia] was dissolved in 2% dimethyl sulfoxide, 0.1 M HCl, 25% (2-Hydroxypropyl)-β-cyclodextrin solution (Sigma-Aldrich, Castle Hill, NSW, Australia) and saline. The pH was adjusted to seven using 0.1 M NaOH. The 20% alcohol (v/v) solution was prepared using 100% food-grade ethyl alcohol (Recochem, SA, Australia) and filtered water. BrdU (5-BromoUracil deoxyriboside, Sigma-Aldrich) was dissolved in 1% DMSO and 0.1 M phosphate-buffered saline (PBS, pH 7.4).

### Drinking-in-the-Dark (DID) Paradigm

We adapted the Drinking-In-the-Dark (DID) model of binge-like alcohol exposure for a long-term period (17 weeks), as previously described (Rhodes et al., 2005; Patkar et al., 2017; Belmer et al., 2018). Briefly, mice were individually housed in double-grommet cages and given access to one bottle of 20% (v/v) alcohol for a 2 h period (12 pm to 2 pm), 3 h into the dark cycle, Monday to Friday. Two bottles of filtered water were available at all other times. Alcohol was presented in 50 ml, graduated, plastic centrifuge tubes (Corning Centristar, New York, NY, USA) fitted with rubber stoppers and a 2.5-inch stainless-steel sipper tube with double ball bearings. Alcohol bottles were weighed before and after 2 h following presentation, and measurements were taken to the nearest 0.1 gram (g). Mouse weights were measured daily to calculate the g/kg alcohol intake.

### Anxiety-Related Behavior

**Figure 1:**
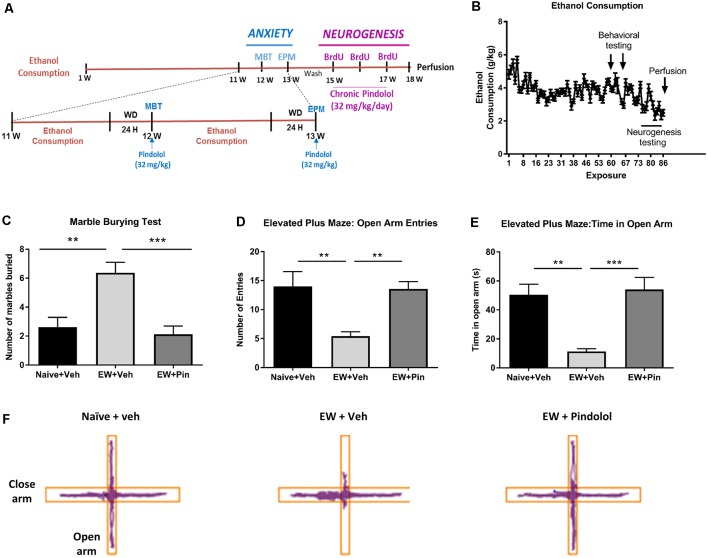
Effects of long-term alcohol consumption levels and pindolol on anxiety-related behavior. Experimental design of the effects of long-term alcohol and pindolol on withdrawal-induced anxiety-like behavior and neurogenesis. Voluntary alcohol intake was allowed in mice for 18 weeks using the Drinking-in-the-Dark (DID) Paradigm paradigm. Anxiety tests; MBP and EPM were carried out in weeks 12 and 13 respectively, where the animals had a 2 h access to alcohol during the weekdays followed by a 24 h withdrawal period before examination of the effects of an acute injection of pindolol (32 mg/kg). These animals were allowed a 2-week drug washout period where they continued to have access to alcohol following which the animals received three acute injections of BrdU (150 mg/kg) in 3 weeks and 2-week chronic injections of pindolol before sacrificing them for the examination of neurogenesis **(A)**. Mean ± SEM alcohol intake (g/kg) for mice in the DID paradigm over 18 weeks or 90 drinking sessions. Anxiety-related tests were carried out at 24 h withdrawal in week 12 (MBT) and week 13 (EPM) while the neurogenesis testing was carried out in weeks 15–18 **(B)**. For the anxiety tests, alcohol-naive mice were treated with vehicle (Naïve+Veh) and alcohol-withdrawn mice with either vehicle (EW+Veh) or pindolol (EW+Pin; **C–F**). MBT shows an increased number of marbles buried by alcohol-withdrawn mice (anxiety-like behavior) which behaved similarly to the naïve controls post pindolol pre-treatment **(C)**. In the EPM, alcohol-withdrawn mice show a reduced number of entries in the open arm of the EPM (anxiety-like behavior) which is altered by pindolol pre-treatment **(D)**. Also, alcohol-withdrawn mice spend less time in open arm of the EPM (anxiety-like behavior) which is reduced by pindolol pre-treatment (**E**). Pindolol (32 mg/kg, i.p.) was administered 30 min prior to a 20 min (MBT) or 5 min (EPM) testing period. Data are presented as mean ± SEM; *n* = 5–6 mice/group. **p < 0.01; ***p < 0.001 (one-way ANOVA followed by Bonferroni’s *post hoc* analysis). Representative tracks of the ambulation of the animals in the open arm vs. close arm of the EPM **(F)**.

Anxiety-like behavior following 24 h alcohol withdrawal was tested on the MBT and the elevated plus-maze (EPM) test. Both tests were conducted during two separate weeks following 12 weeks of drinking (Figure 1) on the same cohort of animals. Briefly, after 12 weeks of alcohol intake, MBT and EPM testing were carried out on two consecutive Sundays in week 12 and week 13 respectively, where the animals had access to alcohol for 2 h during the weekdays and following a 24 h alcohol withdrawal period on a Saturday. On the Sunday, the animals received an acute injection of pindolol (32 mg/kg), 30 min prior to testing them on either a 20 min (MBT) or 5 min (EPM) session (Figure 1, top left and lower panel). Pindolol (32 mg/kg) was chosen since this was the highest effective dose in reducing alcohol intake that did not show the presence of any non-specific effects in mice. Also, pindolol (32 mg/kg) showed no effects on alcohol metabolism or alcohol clearance in the animals as revealed by the loss of righting reflex (LORR) test (Patkar et al., 2017). The alcohol withdrawn mice received either pindolol; EW+Pin (32 mg/kg, i.p. *n* = 5–6,) or vehicle; EW+Veh (*n* = 5–6). The age-matched alcohol naïve water controls received vehicle; Naïve+Veh (*n* = 5–6) to quantify basal anxiety-like behavior.

#### MBT

MBT was performed in novel individual plastic cages (21 × 38 × 14 cm) containing 5-cm thick sawdust bedding. Ten glass marbles (diameter 10–12 mm) were arranged on the bedding evenly spaced in two rows of five marbles. After 20 min, the number of unburied marbles was counted by two experimenters blind to the treatments. A marble-covered at least two-thirds (2/3) of its size by sawdust was considered as “buried.”

#### EPM

EPM was carried out in an apparatus consisting of four arms (35 cm × 5 cm), elevated 50 cm above the floor. The closed arms were enclosed with 40 cm high walls. The experiment was conducted for 5 min, with initial mouse placement in the center. The number of entries and time spent in each arm was recorded using ANY-maze tracking software (Stoelting, IL, USA).

### Neurogenesis Testing

For the neurogenesis testing, the same animals were allowed a 2-week drug washout period following the behavioral testing where they continued to have access to alcohol. These animals received three injections of BrdU (3 × 150 mg/kg, i.p.) over a period of 2 weeks and 2-week chronic injections of pindolol (32 mg/kg, i.p.; Figure 1, top right) only after the daily 2 h drinking period to ensure that the treatment does not interfere with their alcohol consumption. The alcohol-consuming mice received either pindolol; EtOH+Pin (32 mg/kg, i.p. *n* = 5–6,) or vehicle; EtOH+Veh (*n* = 5–6). The age-matched alcohol naïve water controls received vehicle; Naïve+Veh (*n* = 5–6). The animals were allowed another week of alcohol drinking DID sessions after the last BrdU injection before perfusion with 4% paraformaldehyde. The brains were postfixed overnight in at 4°C and sliced into 30 micron-thick coronal sections. After 3 thorough washes in PBS, slices were immersed in ice-cold 10% alcohol-PBS solution for 5 min. After three washes in citrate buffer solution (10 mM citrate buffer, 0.05% Tween 20, pH 6.0) at room temperature, sections were incubated in a 37°C pre-heated citrate buffer solution and placed at 95°C for 20 min, rinsed three times in PBS and incubated overnight in blocking solution (4% normal goat serum-NGS, 1% bovine serum albumin- BSA, 0.3% Triton X100 and 0.05% Tween 20). Sections containing the dorsal hippocampus (Bregma −1.5 mm to −2 mm) were incubated overnight at 4°C with primary antibodies: rabbit anti-DCX (Abcam #18723, 1:500); mouse anti-Ki67 (BD bioscience #550609, 1:20) and rat anti-BrdU (Abcam #6326, 1:200) and with corresponding secondary antibodies, for 2 h at room temperature: goat anti-rabbit-Alexa488; goat anti-mouse-CY5; goat anti-rat-Biotin (Thermo Fisher Scientific, 1:500) and 30 min at room temperature with Streptavidin-Cy3 (Thermo Fisher Scientific, 1:1,000). The sections were mounted in Prolong gold antifade mounting media with DAPI (Thermo Fisher Scientific).

### Imaging and Analysis

Coronal sections of 3–4 dentate gyri at visually similar Bregmas (−1.8 ± 0.3 mm) per animal (*n* = 5–6 animals/group; *N* = 3 groups; total of 19–20 images/group) were imaged on a Nikon/Spectral Spinning Disk confocal microscope in mosaics using a 40× oil-immersion objective (NA 1.35), with a z-step of 0.5 μm. Four-channel mosaic images (.nd2) were deconvolved using Huygens professional v16.10 (Scientific Volume Imaging, Netherlands) with iteration number set at 100, quality threshold at 0.001, signal to noise ratio at 15 for the four channels and converted in .tif for subsequent quantification in Neurolucida 360 (MBF Bioscience). Representative images were taken on an Olympus FV3000, using a 40× oil-objective (NA 1.40), 2.0× zoom and 0.5 z-step. Our previous publication (Belmer et al., 2018) employs the same methodology (antibodies and IHC protocol) as our recent work in which we used validated antibodies, at dilutions recommended by the supplier. Counting of BrdU^+^, DCX^+^, Ki67^+^ and BrdU^+^/Ki67^±^/DCX^±^ cells was performed using a stereology-based approach by an experimenter blind to the treatment on Neurolucida 360 software by setting the reference plane to the first stack and navigating through each stack to the last stack and then, back to the first stack while using the optical dissector function in X and/or Y planes to confirm each counted cell. Only the middle 15 μm of each 30 μm section was imaged to avoid potential double-counting of cells on the adjacent section. For each section, the density of counted cells was normalized to the volume of granular cell layer (GCL) labelled with DAPI in each group using the following formula: density (per μm^3^) = (Number of cells per DG)/(area of GCL in μm^2^ *x* z-depth of the section in μm). Data were then expressed per mm^3^ as follows: density (per mm^3^) = Density (per μm^3^) × 10^9^, then averaged per animal and plotted as mean ± SEM for each group. Overall, one-way ANOVA analysis revealed that the volume of the GCL layer sampled for each group was not significantly different (*F*_(2,56)_ = 0.1238; *p* = 0.8838, Supplementary Figure S1B).

### Statistics

GraphPad Prism 7 (Graph Pad Software Company, CA, USA) was used for all statistical analyses. After determining the normal distribution of each data (Supplementary Table S1), comparisons between groups were statistically analyzed using one-way or two-way ANOVA followed by a Bonferroni multiple comparison *post hoc* test. A comparison of the distribution of BrdU-co-labeled cells was performed using the Chi^2^ test. A *p-value* < 0.05 was considered significant. All values are expressed as the mean ± SEM.

## Results

### Pindolol Reduces Withdrawal-Induced Anxiety-Like Behavior Following Long-Term Alcohol Intake

The DID paradigm produced stable alcohol intake levels (3.69 ± 0.69 g/kg) over a period of 18 weeks (Figure 1B and Supplementary Figure S1A). To evaluate the effects of pindolol on emotional deficits following long-term alcohol exposure, we tested it on withdrawal-induced anxiety-like behavior in the MBT and the elevated plus-maze test (EPM) following 12 and 13 weeks of alcohol intake respectively (Figures 1C–F).

In the MBT, a one-way ANOVA analysis revealed a significant main effect of pindolol treatment on the number of marbles buried (*F*_(2,21)_ = 12.40, ***p = 0.0003, Figure 1C). Bonferroni’s *post hoc* analysis further showed that the EW+Veh group had elevated anxiety-like behavior as compared to their alcohol-naïve counterparts (Naïve+Veh; **p = 0.0019) and pindolol treatment significantly reduced this behavior (***p = 0.0005) in the EW+Pin group, to similar levels as seen in the Naïve+Veh group (*p* > 0.99; Figure 1C).

In the EPM, a one-way ANOVA analysis revealed a significant main effect of pindolol treatment on open arm entries (*F*_(2,18)_ = 8.043, **p = 0.0032) and time spent in open arm (*F*_(2,18)_ = 13.18, ***p = 0.0003; Figures 1D–F). Bonferroni’s *post hoc* analysis further showed that the EW+Veh group had elevated anxiety-like behavior as compared to their alcohol-naïve counterparts (Naïve+Veh) as demonstrated by reduced open arm entries (**p = 0.0067, Figure 1D) and time spent on the open arm (**p = 0.0015, Figure 1E) and pindolol treatment significantly reduced this behavior (Open arm entries; **p = 0.01 and Time spent in open arm; ***p = 0.0006) in the EW+Pin group, to similar levels as seen in the Naïve+Veh group (Open arm entries; *p* > 0.99 and Time spent in open arm; *p* > 0.99). There was no main effect of treatment or between treatment groups in the closed arm entries amongst the three groups (*F*_(2,18)_ = 1.499, *p* = 0.2499). Representative tracks of animal ambulation within the EPM are shown in Figure 1F.

### Chronic Pindolol Treatment Attenuates Neurogenic Deficits of Long-Term Alcohol Exposure

Following the behavioral testing, the mice were allowed a 2-week drug wash-out period following which the effects of pindolol on neurogenic deficits of long-term alcohol exposure were evaluated. For this, we tested the effect of a 2-week chronic pindolol treatment (32 mg/kg/day) on the marker of newborn cells (BrdU), the expression of the cell proliferation marker (Ki67) and the marker of immature neurons (doublecortin; DCX) in the hippocampal DG of mice continuously drinking alcohol for 18 weeks in DID paradigm (Table 1).

**Table 1:** Table showing the nomenclature used to describe cells at different stages of neurogenesis marked by the expression of specific markers.

Cell Markers	Nomenclature
BrdU^+^	Newborn Cells
BrdU^+^ Ki67^+^ DCX^−^	Newborn Proliferating Cells
BrdU^+^ Ki67^+^ DCX^+^	Newborn Proliferating Immature Neurons
BrdU^+^ KI67^−^ DCX^+^	Newborn Non-Proliferating Immature Neurons
KI67^+^	Proliferating Cells
DCX^+^	Immature Neurons

**Figure 2:**
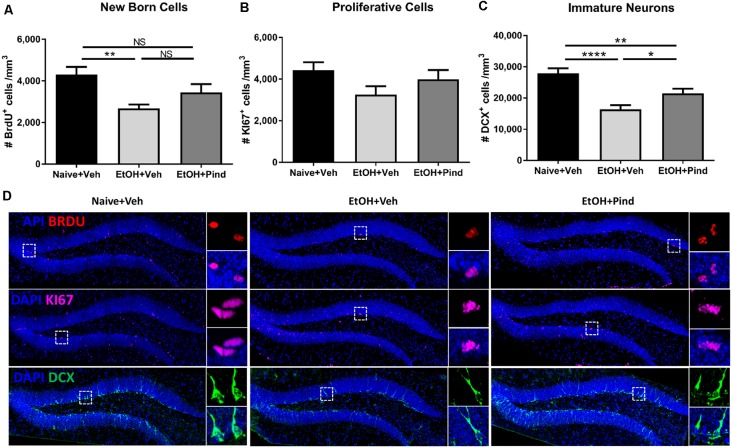
Effects of pindolol on alcohol-induced deficits on markers of neurogenesis in mice. After 12 weeks of alcohol intake, mice were assigned to receive either pindolol (EtOH+Pin) or vehicle (EtOH+Veh). The alcohol naïve mice were assigned to receive a vehicle (Naïve+Veh). All mice received three injections of BrdU/week (150 mg/kg, i.p.) for 3 weeks which was administered at the end of the drinking session on Monday. Following their routine alcohol intake period of 2 h, the EtOH+Pin group received chronic pindolol (32 mg/kg/day), the EtOH+Veh group received chronic vehicle and the Naïve+Veh group received chronic vehicle injections for 2 weeks. The animals were allowed 1 week of alcohol intake after the last BrdU injection before sacrificing, *n* = 5–6 animals/group; six dentate gyri/animal. Long-term alcohol drinking reduces the density of BrdU-immunoreactive (BrdU^+^) cells in the dentate gyrus (DG) of the hippocampus and chronic pindolol treatment has no effect on this impairment **(A)**. The density of Ki67-immunoreactive cells (Ki67^+^) is not affected by alcohol consumption or pindolol treatment **(B)**. Long-term chronic alcohol decreases the density of DCX-immunoreactive (DCX^+^) immature neurons in the DG, and this reduction was only partially prevented by a chronic pindolol treatment, as compared to alcohol naive mice treated with vehicle **(C)**. Data are presented as mean density of cells per mm^3^ of granular layer ± SEM; *n* = 5–6 mice/group, 3–4 dentate gyri/mouse. *p < 0.05, **p < 0.01; ****p < 0.0001 (one-way ANOVA followed by Bonferroni’s *post hoc* analysis). Representative confocal micrographs for the different markers (DAPI+BrdU; DAPI+KI67 and DAPI+DCX; scale bar = 300 μm) are presented for different treatment groups (naïve+Veh, EtOH+veh and EtOH+Pind; **D)**. For each micrograph, a higher magnification view of the dash box (scale bar = 15 μm) is presented on the right. ns = not significant, *p* > 0.05.

A one-way ANOVA analysis revealed a main effect of alcohol exposure on the density of BrdU^+^ cells (*F*_(2,56)_ = 5.995, **p = 0.0044). Bonferroni’s *post hoc* analysis showed that long-term alcohol consumption significantly reduced the density of BrdU^+^ cells in alcohol-exposed mice as compared to naïve controls (**p = 0.0031, Figure 2A) and pindolol treatment had no effect in alleviating this reduction (ns: *p* = 0.2347).

One-way ANOVA analysis revealed no main effect of pindolol treatment on the expression of the cell proliferation marker: Ki67 (*F*_(2,56)_ = 2.032, *p* = 0.1406, Figure 2B) in the groups. However, one-way ANOVA revealed a main effect of alcohol exposure on the density of immature DCX^+^ neurons (*F*_(2,56)_ = 15.56, ****p < 0.0001). Bonferroni’s *post hoc* analysis further showed that long-term alcohol consumption significantly reduced the density of DCX^+^ cells in the DG of alcohol-exposed mice as compared to naïve controls (****p < 0.0001) and pindolol treatment significantly blocked this reduction (*p = 0.0451, Figure 2C), but could only partially reverse the effects of alcohol as compared to the alcohol naïve controls (**p = 0.0092). Representative confocal micrographs of BrdU, Ki67 and DCX labeling are shown in Figure 2D.

**Figure 3:**
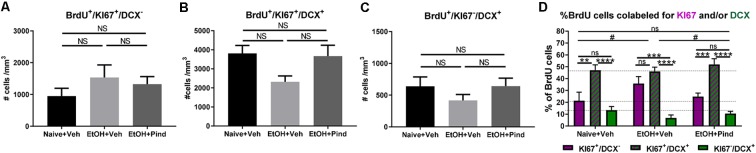
Effects of pindolol on alcohol-induced impairments in the proportion of newborn proliferating cells, newborn proliferating immature neurons and newborn non-proliferating immature neurons. The density of BrdU^+^/Ki67^+^/DCX^−^ newborn proliferating cells is not affected by alcohol consumption or pindolol pre-treatment **(A)**. Long-term alcohol drinking or pindolol also have no effect on the density of BrdU^+^/Ki67^+^/DCX^+^ newborn proliferating immature neurons in the DG, however a strong trend towards reduction is seen in the alcohol group as compared to alcohol naïve controls **(B)**. The density of BrdU^+^/Ki67^−^/DCX^+^ newborn non-proliferating immature neurons is not affected by alcohol consumption or pindolol pre-treatment **(C)**. **(A–C)** Data are presented as mean density of cells per mm3 of granular layer ± SEM; *n* = 5–6 mice/group, 5–6 dentate gyri/mouse (one-way ANOVA followed by Bonferroni’s *post hoc* analysis). Long-term alcohol consumption impacts the proportion of newborn proliferating cells, newborn proliferating immature neurons, and newborn non-proliferating immature neurons as compared to alcohol naïve controls which is restored by chronic pindolol treatment **(D)**. Data are presented as mean percentage of BrdU^+^ cells ± SEM. **p < 0.01; ***p < 0.001, ****p < 0.0001 (two-way ANOVA with Bonferroni’s *post hoc* test). ^#^*p* < 0.05 (Chi-squared distribution analysis). ns = not significant, *p* > 0.05.

We then looked at the density of newborn proliferating cells (BrdU^+^/Ki67^+^/DCX^−^), newborn proliferating immature neurons (BrdU^+^/Ki67^+^/DCX^+^) and newborn non-proliferating immature neurons (BrdU^+^/Ki67^−^/DCX^+^) in the DG of alcohol-exposed mice to see if the alcohol treatment or pindolol administration had any effect in the population of these cells. One-way ANOVA revealed no effect of pindolol treatment or between-group differences in the density of newborn proliferating cells (BrdU^+^/Ki67^+^/DCX^−^; *F*_(2,56)_ = 0.9435, *p* = 0.3953, Figure 3A) or newborn non-proliferating immature neurons (BrdU^+^/Ki67^−^/DCX^+^; *F*_(2,60)_ = 1.073, *p* = 0.3484, Figure 3C). However, there was a significant main effect in the density of new-born proliferating immature neurons (BrdU^+^/Ki67^+^/DCX^+^; *F*_(2,56)_ = 3.395, *p = 0.0406, Figure 3B) between the treatment groups. Bonferroni’s *post hoc* analysis, however, showed a strong trend but no significant effect of alcohol in reducing the number of new-born immature neurons as compared to the alcohol naïve controls (ns: *p* = 0.0684) and pindolol had no effect in this group (ns: *p* = 0.1076).

A comparative two-way ANOVA analysis of the percentage of BrdU^+^ cells that were either proliferating (BrdU^+^/Ki67^+^/DCX^−^) or proliferating immature neurons (BrdU^+^/Ki67^+^/DCX^+^) or non-proliferating immature neurons (BrdU^+^/Ki67^−^/DCX^+^) in the three treatment groups revealed no effect of treatment (*F*_(2,141)_ = 0.2482, *p* = 0.78) but a significant effect of the cell-type *F*_(2,141)_ = 67.89, ****p < 0.0001 (Figure 3D), with no significant treatment × cell-type interaction (*F*_(4,141)_ = 2.025, *p* = 0.094057). Bonferroni’s *post hoc* analysis of BrdU-co-labeled cells revealed a higher proportion of proliferating immature neurons (BrdU^+^/Ki67^+^/DCX^+^, 47.09 ± 4.5%) compared to proliferating cells (BrdU^+^/Ki67^+^/DCX^−^, 21.34 ± 7.2%, **p = 0.002) and non-proliferating immature neurons (BrdU^+^/Ki67^−^/DCX^+^, 13.42 ± 2.9%, ****p < 0.0001) in the Naïve+Veh group, with no difference between (BrdU^+^/Ki67^+^/DCX^−^) and (BrdU^+^/Ki67^−^/DCX^+^; ns, *p* > 0.99). In the EtOH+Veh group, however, there was a higher proportion of proliferating immature neurons (BrdU^+^/Ki67^+^/DCX^+^, 46.05 ± 3.5%) than non-proliferating immature neurons (BrdU^+^/Ki67^−^/DCX^+^, 6.84 ± 2.5%; ****p < 0.0001), but not different from the proportion of proliferating cells (BrdU^+^/Ki67^+^/DCX^−^, 35.75, ns, *p* > 0.99), with the proportion of proliferating cells (BrdU^+^/Ki67^+^/DCX^−^) significantly higher than non-proliferating immature neurons (BrdU^+^/Ki67^−^/DCX^+^; ***p = 0.001). Interestingly, we found that pindolol treatment significantly prevented this impairment and restored a higher proportion of proliferating immature neurons (BrdU^+^/Ki67^+^/DCX^+^, 51.96 ± 4.7%) in the EtOH+Pind group, as compared to proliferating cells (BrdU^+^/Ki67^+^/DCX^−^, 24.88 ± 2.8%, ***p = 0.0002) and non-proliferating immature neurons (BrdU^+^/Ki67^−^/DCX^+^, 10.50 ± 1.8%, ****p < 0.0001), and eliminated the difference between the proportion of proliferating (BrdU^+^/Ki67^+^/DCX^−^) and (BrdU^+^/Ki67^−^/DCX^+^; ns, *p* = 0.75; Figure 3D and Supplementary Table S2). Furthermore, Chi^2^ analysis revealed that ethanol (EtOH+Veh) significantly changes the distribution of BrdU co-labeled cells compared to control (Naïve+Veh; *p = 0.012 ; Chi^2^, *df* = 8.81, 2). Interestingly, pindolol treatment in ethanol exposed animals (EtOH+Pind) prevented this impairment (*p = 0.045, Chi^2^, *df* = 6.19, 2) and restored the distribution of BrdU co-labeled cells to a level similar to control animals (Naïve+Veh; ns, *p* = 0.75; Chi^2^, *df* = 0.59, 2).

## Discussion

Anxiety, depression and other mood disorders, when untreated, often present with comorbid substance abuse (Penick et al., 1988; Ross, 1995; Hall et al., 1999) and several research labs employing animal models of affective and addictive disorders report concomitant dysregulation of adult hippocampal neurogenesis in these animals (Nixon and Crews, 2004; He et al., 2005; Mineur et al., 2007; Petrik et al., 2012). Previous studies have shown that treatment of mood disorders with SSRIs requires improvements in hippocampal neurogenesis to mediate their antidepressant effects and blockade of enhancements in neurogenesis reduces the efficacy of SSRIs (Santarelli et al., 2003; Hill et al., 2015; Tunc-Ozcan et al., 2019). This suggests that despite the complexity of multiple neurobiological processes controlling behavior, alterations in hippocampal neurogenesis could be linked as the major cause of precipitation of negative affective states and substance abuse, including alcohol.

Neural stem cells from the subgranular zone of the hippocampal DG are maintained in a quiescent stage before encountering proliferation signals from several environmental and neurotrophic factors (Carleton et al., 2003). These proliferating cells differentiate into newborn neurons and mature through a series of maturation steps before integrating into the hippocampal network (Zhao et al., 2008) where they facilitate various functions, such as learning, memory and mood regulation (Gould, 1999; Santarelli et al., 2003; Lezi et al., 2014). Previous studies have shown that alcohol interferes with normal neurogenesis which is thought to contribute to the reduced hippocampal volume seen in human adolescents with AUDs (Nagel et al., 2005; Medina et al., 2007). In the present study, we examined the effect of long-term binge alcohol intake in adolescent mice on neurogenesis and emotional behavior at adulthood. Furthermore, since our previous reports suggest the efficacy of the antihypertensive drug, pindolol, in attenuating consumption in adolescent drinking behavior (Patkar et al., 2017, 2019), we also tested its effects on the emotional and neurogenic consequences of alcohol in this model. We have modified the well-established DID protocol in mice to enable long-term voluntary alcohol consumption which facilitates stable rodent alcohol drinking levels and high blood alcohol concentrations. The long term DID model of rodent alcohol intake also closely mimics human-like voluntary binge drinking to best reflect the neurobiological and emotional alterations of alcohol on the maturation of the adolescent brain.

Research in the past has utilized varying degrees of severity of alcohol administration paradigms in rodents ranging from acute to chronic intragastric gavage methods to study the effects of alcohol on neurogenesis. Acute (Crews et al., 2006) and chronic alcohol (Morris et al., 2010) forced exposure retards neurogenesis as measured by a reduction in the expression of the marker of immature neurons, DCX. This reduction in neurogenesis is facilitated by the negative effects of alcohol on cell division whereby, the number of newborn cells is drastically reduced as evidenced by decreased integration of synthetic nucleoside BrdU (Crews et al., 2006; Morris et al., 2010). In the present study, we employed a modified BrdU administration protocol which enabled us to label discrete populations of newborn neuronal cells at different stages of neurogenesis, all at the same time point of animal sacrifice. The first (Week 15), second (Week 16) and third (Week 17) BrdU injection coupled with the time of animal sacrifice (1 week after the last BrdU injection), has enabled the identification of newborn immature neurons (BRDU^+^; KI67^−^; DCX^+^), proliferating immature neurons (BRDU^+^; KI67^+^; DCX^+^) and newborn proliferating cells (BRDU^+^; KI67^+^, DCX^−^), although this experimental procedure did not allow for precise dating of the birth of these cells.

Using this approach, we show that prolonged alcohol consumption affects neurogenesis by reducing either the proliferation, maturation or survival of new-born cells. This observation is also in agreement with previous studies employing acute and chronic models of forced alcohol exposure. suggesting that alcohol, irrespective of the mode of delivery, may intervene at the G1 phase of the cell cycle, arresting cells and thereby causing a reduction in the number of cells entering the S phase where BrdU gets incorporated. This theory is also supported by the observed lack of change in the density of Ki67^+^ cells since Ki67 is expressed throughout the cell cycle and this density is likely to remain the same irrespective of the phase of cell harvest (Morris et al., 2010; Khatri et al., 2018). Interestingly, some reports suggest that chronic alcohol affects Ki67^+^ proliferating cells in adults more than adolescents rats (Morris et al., 2010), however we did not observe this in the present study possibly because of the differences in the species of rodents and/or models of the alcohol delivery.

More interestingly, long-term alcohol studies (11 months) in adolescent monkeys show a reduction in Ki67^+^ cells at proliferating and migrating stage before complete neuronal differentiation, implying that alcohol exposure duration in key stages of adolescence may have different impacts on neurogenesis (Taffe et al., 2010). However, since long-term alcohol abuse is reported to cause long-lasting neurobiological and behavioral changes that manifest majorly in withdrawal, the examination of neurogenesis during abstinence would highlight the effects of prolonged exposure of alcohol to the developing adolescent brain. It would be interesting to compare the validity and the effect of the long-term model with previous reports that suggest an increase in density of newborn and proliferating cells at 7 days and a population of immature neurons at 14 days in withdrawal, respectively (Nixon and Crews, 2004). Furthermore, we also report that the proportion of BrdU^+^ cells that were either proliferating (BrdU^+^/Ki67^+^/DCX^−^) or differentiated into proliferating immature neurons (BrdU^+^/Ki67^+^/DCX^+^) or non-proliferating immature neurons (BrdU^+^/Ki67^−^/DCX^+^) is altered by alcohol. This suggests that in addition to reducing the density of new-born cells, alcohol may alter the proportion of new-born proliferating cells (BrdU^+^/Ki67^+^/DCX^−^) transitioning into new-born immature neurons (BrdU^+^/Ki67^+^/DCX^−^). However, the current experimental design can not explain whether this alteration in transition is due to deficits in the neuronal differentiation process itself, changes in the fate of differentiation of the cells (astrocytes, oligodendrocytes) or reduced survival of the immature neurons.

The reduction in the density BrdU^+^ cells may be due to impaired survival of these cells immediately following differentiation or after losing the expression of Ki67 (BrdU^+^/Ki67^−^/DCX^+^). Analysis of this specific population of cells (BrdU^+^/Ki67^−^/DCX^+^) revealed no difference between the naïve and alcohol groups (Figure 3C) suggesting that alcohol may directly affect the survival of BrdU^+^/Ki67^+^/DCX^+^ cells. This effect of alcohol is also reflected in the reduced numbers of total DCX^+^ cells (Figure 2C). Indeed, it is also possible that alcohol exposure may affect the ability of newborn proliferating cells to acquire either neuronal or glial specific signatures in the differentiation phase (Nixon and Crews, 2004; He et al., 2005; Morris et al., 2010), however the use of other, more stage-specific differentiation markers is warranted to confirm this. In addition, adult hippocampal neurogenesis involves the proliferation of neural stem cells which results in the generation of pluripotent transit-amplifying progenitor cells (TAPs; Potten and Loeffler, 1990). These cells are highly proliferative, differentiate rapidly and have the ability to generate a large number of mature differentiating cells from a relatively low population of progenitor cells (Carleton et al., 2003; Kempermann et al., 2004). Since TAPs remain quiescent for long periods (Doetsch et al., 2002) before experiencing a burst in differentiation, it is also possible that alcohol reduces the number of TAPs which are BrdU^+^ resulting in the observed changes in neurogenesis.

Interestingly, chronic pindolol treatment for the last 2 weeks of an 18-week alcohol exposure period rescued the impairments in the density of immature neurons and restored the normal proportion of newborn proliferating cells and newborn immature neurons. Pindolol, however, had no effect in recovering the impairments of alcohol on the density of newborn cells. Pindolol has a dual mechanism of action, having an antagonistic effect on β_1_/β_2_ adrenoreceptors and a partial agonistic effect on 5-HT1A/1B receptors. In a separate study using the same long-term alcohol intake mouse model, the selective 5-HT1A partial agonist tandospirone, increased the density of newborn cells, proliferative cells and immature neurons (Belmer et al., 2018), suggesting an effect at either the cell division, proliferating, differentiating or all phases of neurogenesis. Chronic treatment of tandospirone also caused a much higher increase in the density of BrdU^+^ and DCX^+^ cells as compared to chronic pindolol treatment (Belmer et al., 2018) in long-term alcohol-consuming mice. It is possible that since pindolol is a weak partial agonist at 5-HT1A receptors compared to tandospirone, the latter may have a stronger effect in restoring altered neurogenesis. This suggests that, despite the complex pharmacology of pindolol, the similitudes between the effects of pindolol and tandospirone implicates a strong role of 5-HT1A receptors in mediating the effects of alcohol on emotional and neurogenic deficits.

Drugs that modulate 5-HT and NE signaling including selective reuptake inhibitors, agonists and antagonists have been shown to modulate hippocampal neurogenesis and improve emotional behavior in animals (Santarelli et al., 2003; Banasr et al., 2004; Jhaveri et al., 2010; Silberman et al., 2012; Mori et al., 2014; Nautiyal et al., 2016; Belmer et al., 2018). Therefore, it is not completely surprising that pindolol, which can modulate NE and 5-HT signaling, had an effect on anxiety-like behavior in mice. Clinical and preclinical studies in the past have shown that pindolol accelerates the onset of the therapeutic effects of SSRI treatment in humans presumably *via* its activity on 5HT receptors (Isaac, 2004; Sokolski et al., 2004). Similarly, tandospirone also produced robust effects on withdrawal-induced anxiety-like behavior in the long-term mouse model of alcohol intake (Belmer et al., 2018). It may be possible that pindolol’s serotonergic pharmacology may contribute to its effects on emotional behavior and neurogenesis (Sharp et al., 1993; Hjorth et al., 1996). A recent report demonstrates the crucial requirement of the activity of newborn hippocampal neurons to mediate the effects of SSRIs (Tunc-Ozcan et al., 2019). The blockade of the activity of these neurons reduced the antidepressant effect of SSRI, fluoxetine. Since we demonstrate that both pindolol and tandospirone reduce the deficits of alcohol on the differentiation of immature DCX neurons from new-born cells, it is likely that this may be sufficient to elicit the therapeutic effects on emotional and alcohol seeking behavior of these treatment drugs.

## Ethics Statement

This study was carried out in accordance with the recommendations of National Health and Medical Research Council (NHMRC) guidelines to promote the well-being of animals used for scientific purposes and the Australian code for the care and use of animals for scientific purposes. The protocol was approved by the Queensland University of Technology Animal Ethics Committee and the University of Queensland Animal Ethics Committee. Approval number QUT/053/18.

## Author Contributions

OP, AB and SB were responsible for the study concept and design. AB and OP carried out behavioral animal experiments. AB performed the immunohistochemistry experiments. OP acquired the images and drafted the manuscript. KB, AJ and AB analyzed the data and interpreted the findings. AB, SB and OP reviewed the manuscript. All authors have critically reviewed the content and approved the final version for submission.

## Conflict of Interest

The authors declare that the research was conducted in the absence of any commercial or financial relationships that could be construed as a potential conflict of interest.

## Supplementary Material

FIGURE S1 **(A)** Stability of ethanol intake along the last 6-weeks of DID, during behavioral and neurogenesis testing at selected time-points, at week 12, 13, 15 and 18. No significant changes were observed in each treatment group (two-way ANOVA with repeated measures and Bonferroni’s multiple comparison. Effect of time: *F*_(3,42)_ = 1.823, *P* = 0.1577). Effect of treatment: *F*_(1,14)_ = 0.3692, *p* = 0.5532). **(B)** Volume of granular cell layer samples for each group in the neurogenesis experiment. No significant change was observed between groups (*F*_(2,56)_ = 0.1238, *P* = 0.8838). ns = not significant, *p* > 0.05.

TABLE S1 Statistical analysis showing normality of cell counts using three different tests. Results of the normality tests are highlighted in yellow. ns, non-significant; EtOH, ethanol; Pind, pindolol; Veh, vehicle.

TABLE S2 Two-way ANOVA analysis of BrdU cells co-labeling (Figure 3D): multiple comparison using Bonferroni *Post hoc* Analysis. Significant results are highlighted in gray.
