# Supplementary figures and images for: Pindolol Rescues Anxiety-Like Behavior and Neurogenic Maladaptations of Long-Term Binge Alcohol Intake in Mice
Source: Front Behav Neurosci. 2019 Nov 29;13:264. doi: 10.3389/fnbeh.2019.00264 (PMC6895681; doi:10.3389/fnbeh.2019.00264)

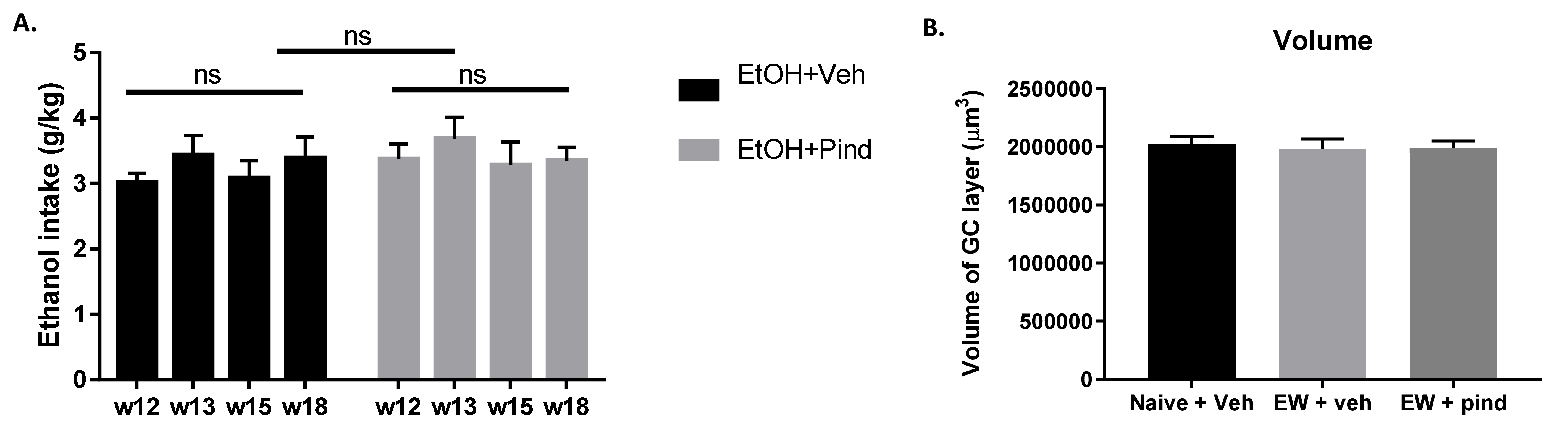

Supplement: FIGURE S1 — (A) Stability of ethanol intake along the last 6-weeks of DID, during behavioral and neurogenesis testing at selected time-points, at week 12, 13, 15 and 18. No significant changes were observed in each treatment group (two-way ANOVA with repeated measures and Bonferroni’s multiple comparison. Effect of time: F(3,42) = 1.823, P = 0.1577). Effect of treatment: F(1,14) = 0.3692, p = 0.5532). (B) Volume of granular cell layer samples for each group in the neurogenesis experiment. No significant change was observed between groups (F(2,56) = 0.1238, P = 0.8838). ns = not significant, p > 0.05. [file Image_1.TIF]
