# Supplementary material for: Pindolol Rescues Anxiety-Like Behavior and Neurogenic Maladaptations of Long-Term Binge Alcohol Intake in Mice
Source: Front Behav Neurosci. 2019 Nov 29;13:264. doi: 10.3389/fnbeh.2019.00264 (PMC6895681; doi:10.3389/fnbeh.2019.00264)
Supplement: TABLE S1 — Statistical analysis showing normality of cell counts using three different tests. Results of the normality tests are highlighted in yellow. ns, non-significant; EtOH, ethanol; Pind, pindolol; Veh, vehicle. [file Table_1.DOCX]

**Supplementary Table 1: Statistical analysis showing normality of cell counts using 3 different tests.** Results of the normality tests are highlighted in yellow, ns= non-significant, EtOH= ethanol, Pind= pindolol, Veh= vehicle.

| **DCX +** | **EtOH+Pind** | **EtOH+Veh** | **Naive+Veh** |
| --- | --- | --- | --- |
| Mean | 21523 | 16400 | 27922 |
| Std. Deviation | 6506 | 5894 | 6952 |
| Std. Error of Mean | 1455 | 1318 | 1595 |
| D'Agostino & Pearson normality test |  |  |  |
| K2 | 1.296 | 5.199 | 0.3605 |
| P value | 0.5232 | 0.0743 | 0.8351 |
| Passed normality test (alpha=0.05)? | Yes | Yes | Yes |
| P value summary | ns | ns | ns |
| Shapiro-Wilk normality test |  |  |  |
| W | 0.9473 | 0.9251 | 0.9845 |
| P value | 0.3277 | 0.1243 | 0.9817 |
| Passed normality test (alpha=0.05)? | Yes | Yes | Yes |
| P value summary | ns | ns | ns |
| KS normality test |  |  |  |
| KS distance | 0.1635 | 0.123 | 0.09238 |
| P value | >0.1000 | >0.1000 | >0.1000 |
| Passed normality test (alpha=0.05)? | Yes | Yes | Yes |
| P value summary | ns | ns | ns |
|  |  |  |  |
| **BrdU+** | **EtOH+Pind** | **EtOH+Veh** | **Naive+Veh** |
| Mean | 3446 | 2683 | 4306 |
| Std. Deviation | 1789 | 830.7 | 1599 |
| Std. Error of Mean | 400.1 | 185.7 | 366.8 |
| **D'Agostino & Pearson normality test** |  |  |  |
| K2 | 0.1519 | 0.9749 | 0.6146 |
| P value | 0.9269 | 0.6142 | 0.7354 |
| Passed normality test (alpha=0.05)? | Yes | Yes | Yes |
| P value summary | ns | ns | ns |
| **Shapiro-Wilk normality test** |  |  |  |
| W | 0.9714 | 0.9398 | 0.9799 |
| P value | 0.7844 | 0.2377 | 0.9412 |
| Passed normality test (alpha=0.05)? | Yes | Yes | Yes |
| P value summary | ns | ns | ns |
| **KS normality test** |  |  |  |
| KS distance | 0.1189 | 0.113 | 0.1009 |
| P value | >0.1000 | >0.1000 | >0.1000 |
| Passed normality test (alpha=0.05)? | Yes | Yes | Yes |
| P value summary | ns | ns | ns |
|  |  |  |  |
| **KI67+** | **EtOH+Pind** | **EtOH+Veh** | **Naive+Veh** |
| Mean | 3993 | 3256 | 4428 |
| Std. Deviation | 1981 | 1819 | 1703 |
| Std. Error of Mean | 443 | 406.8 | 390.8 |
| **D'Agostino & Pearson normality test** |  |  |  |
| K2 | 1.191 | 3.274 | 0.4075 |
| P value | 0.5512 | 0.1946 | 0.8157 |
| Passed normality test (alpha=0.05)? | Yes | Yes | Yes |
| P value summary | ns | ns | ns |
| **Shapiro-Wilk normality test** |  |  |  |
| W | 0.9584 | 0.9376 | 0.9771 |
| P value | 0.5116 | 0.2162 | 0.9038 |
| Passed normality test (alpha=0.05)? | Yes | Yes | Yes |
| P value summary | ns | ns | ns |
| **KS normality test** |  |  |  |
| KS distance | 0.1258 | 0.1465 | 0.1082 |
| P value | >0.1000 | >0.1000 | >0.1000 |
| Passed normality test (alpha=0.05)? | Yes | Yes | Yes |
| P value summary | ns | ns | ns |
|  |  |  |  |
| **BrdU+/KI67+** | **EtOH+Pind** | **EtOH+Veh** | **Naive+Veh** |
| Mean | 1324 | 1535 | 950 |
| Std. Deviation | 1062 | 1765 | 1065 |
| Std. Error of Mean | 237.6 | 394.6 | 244.3 |
| **D'Agostino & Pearson normality test** |  |  |  |
| K2 | 2.22 | 2.997 | 1.305 |
| P value | 0.3296 | 0.2235 | 0.5206 |
| Passed normality test (alpha=0.05)? | Yes | Yes | Yes |
| P value summary | ns | ns | ns |
| **Shapiro-Wilk normality test** |  |  |  |
| W | 0.9427 | 0.9087 | 0.8943 |
| P value | 0.2695 | 0.095 | 0.0546 |
| Passed normality test (alpha=0.05)? | Yes | Yes | Yes |
| P value summary | ns | ns | ns |
| **KS normality test** |  |  |  |
| KS distance | 0.1063 | 0.1693 | 0.2072 |
| P value | >0.1000 | >0.1000 | 0.0506 |
| Passed normality test (alpha=0.05)? | Yes | Yes | Yes |
| P value summary | ns | ns | ns |

|  |  |  |  |
| --- | --- | --- | --- |
| **BrdU+/KI67+/DCX+** | **EtOH+Pind** | **EtOH+Veh** | **Naive+Veh** |
| Mean | 1835 | 1159 | 1904 |
| Std. Deviation | 1277 | 697.1 | 913.5 |
| Std. Error of Mean | 285.6 | 155.9 | 209.6 |
| **D'Agostino & Pearson normality test** |  |  |  |
| K2 | 1.959 | 0.7659 | 5.199 |
| P value | 0.3755 | 0.6818 | 0.0743 |
| Passed normality test (alpha=0.05)? | Yes | Yes | Yes |
| P value summary | ns | ns | ns |
| **Shapiro-Wilk normality test** |  |  |  |
| W | 0.9524 | 0.975 | 0.8985 |
| P value | 0.4054 | 0.8551 | 0.0541 |
| Passed normality test (alpha=0.05)? | Yes | Yes | Yes |
| P value summary | ns | ns | ns |
| **KS normality test** |  |  |  |
| KS distance | 0.1327 | 0.1051 | 0.2003 |
| P value | >0.1000 | >0.1000 | 0.0544 |
| Passed normality test (alpha=0.05)? | Yes | Yes | Yes |
| P value summary | ns | ns | ns |

**Supplementary Table S2 : Two-way ANOVA analysis of BrdU cells co-labeling (Fig. 3D):** Multiple comparison using Bonferroni Post hoc Analysis. Significant results are highlighted in grey.

| Bonferroni's multiple comparisons test | Mean Diff. | 95.00% CI of diff. | Significant? | Summary | Adjusted P Value |
| --- | --- | --- | --- | --- | --- |
| Naive+Veh:BrdU+/KI67+/DCX- vs. Naive+Veh:BrdU+/KI67+/DCX+ | -25.76 | -46 to -5.523 | Yes | ** | 0.0020 |
| Naive+Veh:BrdU+/KI67+/DCX- vs. Naive+Veh:BrdU+/KI67-/DCX+ | 7.914 | -11.65 to 27.48 | No | ns | >0.9999 |
| Naive+Veh:BrdU+/KI67+/DCX- vs. EtOH+Veh:BrdU+/KI67+/DCX- | -14.41 | -35.57 to 6.744 | No | ns | >0.9999 |
| Naive+Veh:BrdU+/KI67+/DCX- vs. EtOH+Veh:BrdU+/KI67+/DCX+ | -24.71 | -44.7 to -4.719 | Yes | ** | 0.0033 |
| Naive+Veh:BrdU+/KI67+/DCX- vs. EtOH+Veh:BrdU+/KI67-/DCX+ | 14.5 | -5.273 to 34.27 | No | ns | 0.6512 |
| Naive+Veh:BrdU+/KI67+/DCX- vs. EtOH+Pind:BrdU+/KI67+/DCX- | -3.541 | -24.05 to 16.97 | No | ns | >0.9999 |
| Naive+Veh:BrdU+/KI67+/DCX- vs. EtOH+Pind:BrdU+/KI67+/DCX+ | -30.62 | -50.39 to -10.85 | Yes | **** | <0.0001 |
| Naive+Veh:BrdU+/KI67+/DCX- vs. EtOH+Pind:BrdU+/KI67-/DCX+ | 10.83 | -10.32 to 31.99 | No | ns | >0.9999 |
| Naive+Veh:BrdU+/KI67+/DCX+ vs. Naive+Veh:BrdU+/KI67-/DCX+ | 33.67 | 15.55 to 51.79 | Yes | **** | <0.0001 |
| Naive+Veh:BrdU+/KI67+/DCX+ vs. EtOH+Veh:BrdU+/KI67+/DCX- | 11.35 | -8.475 to 31.17 | No | ns | >0.9999 |
| Naive+Veh:BrdU+/KI67+/DCX+ vs. EtOH+Veh:BrdU+/KI67+/DCX+ | 1.049 | -17.53 to 19.62 | No | ns | >0.9999 |
| Naive+Veh:BrdU+/KI67+/DCX+ vs. EtOH+Veh:BrdU+/KI67-/DCX+ | 40.26 | 21.92 to 58.59 | Yes | **** | <0.0001 |
| Naive+Veh:BrdU+/KI67+/DCX+ vs. EtOH+Pind:BrdU+/KI67+/DCX- | 22.22 | 3.087 to 41.35 | Yes | ** | 0.0081 |
| Naive+Veh:BrdU+/KI67+/DCX+ vs. EtOH+Pind:BrdU+/KI67+/DCX+ | -4.864 | -23.2 to 13.47 | No | ns | >0.9999 |
| Naive+Veh:BrdU+/KI67+/DCX+ vs. EtOH+Pind:BrdU+/KI67-/DCX+ | 36.59 | 16.77 to 56.42 | Yes | **** | <0.0001 |
| Naive+Veh:BrdU+/KI67-/DCX+ vs. EtOH+Veh:BrdU+/KI67+/DCX- | -22.33 | -41.46 to -3.186 | Yes | ** | 0.0076 |
| Naive+Veh:BrdU+/KI67-/DCX+ vs. EtOH+Veh:BrdU+/KI67+/DCX+ | -32.62 | -50.47 to -14.78 | Yes | **** | <0.0001 |
| Naive+Veh:BrdU+/KI67-/DCX+ vs. EtOH+Veh:BrdU+/KI67-/DCX+ | 6.582 | -11.01 to 24.18 | No | ns | >0.9999 |
| Naive+Veh:BrdU+/KI67-/DCX+ vs. EtOH+Pind:BrdU+/KI67+/DCX- | -11.45 | -29.88 to 6.968 | No | ns | >0.9999 |
| Naive+Veh:BrdU+/KI67-/DCX+ vs. EtOH+Pind:BrdU+/KI67+/DCX+ | -38.54 | -56.13 to -20.94 | Yes | **** | <0.0001 |
| Naive+Veh:BrdU+/KI67-/DCX+ vs. EtOH+Pind:BrdU+/KI67-/DCX+ | 2.92 | -16.22 to 22.06 | No | ns | >0.9999 |
| EtOH+Veh:BrdU+/KI67+/DCX- vs. EtOH+Veh:BrdU+/KI67+/DCX+ | -10.3 | -29.87 to 9.274 | No | ns | >0.9999 |
| EtOH+Veh:BrdU+/KI67+/DCX- vs. EtOH+Veh:BrdU+/KI67-/DCX+ | 28.91 | 9.562 to 48.25 | Yes | *** | 0.0001 |
| EtOH+Veh:BrdU+/KI67+/DCX- vs. EtOH+Pind:BrdU+/KI67+/DCX- | 10.87 | -9.23 to 30.97 | No | ns | >0.9999 |
| EtOH+Veh:BrdU+/KI67+/DCX- vs. EtOH+Pind:BrdU+/KI67+/DCX+ | -16.21 | -35.56 to 3.134 | No | ns | 0.2546 |
| EtOH+Veh:BrdU+/KI67+/DCX- vs. EtOH+Pind:BrdU+/KI67-/DCX+ | 25.24 | 4.485 to 46 | Yes | ** | 0.0042 |
| EtOH+Veh:BrdU+/KI67+/DCX+ vs. EtOH+Veh:BrdU+/KI67-/DCX+ | 39.21 | 21.14 to 57.27 | Yes | **** | <0.0001 |
| EtOH+Veh:BrdU+/KI67+/DCX+ vs. EtOH+Pind:BrdU+/KI67+/DCX- | 21.17 | 2.298 to 40.04 | Yes | * | 0.0129 |
| EtOH+Veh:BrdU+/KI67+/DCX+ vs. EtOH+Pind:BrdU+/KI67+/DCX+ | -5.913 | -23.98 to 12.15 | No | ns | >0.9999 |
| EtOH+Veh:BrdU+/KI67+/DCX+ vs. EtOH+Pind:BrdU+/KI67-/DCX+ | 35.54 | 15.97 to 55.12 | Yes | **** | <0.0001 |
| EtOH+Veh:BrdU+/KI67-/DCX+ vs. EtOH+Pind:BrdU+/KI67+/DCX- | -18.04 | -36.67 to 0.5994 | No | ns | 0.0702 |
| EtOH+Veh:BrdU+/KI67-/DCX+ vs. EtOH+Pind:BrdU+/KI67+/DCX+ | -45.12 | -62.94 to -27.3 | Yes | **** | <0.0001 |
| EtOH+Veh:BrdU+/KI67-/DCX+ vs. EtOH+Pind:BrdU+/KI67-/DCX+ | -3.663 | -23.01 to 15.68 | No | ns | >0.9999 |
| EtOH+Pind:BrdU+/KI67+/DCX- vs. EtOH+Pind:BrdU+/KI67+/DCX+ | -27.08 | -45.72 to -8.446 | Yes | *** | 0.0002 |
| EtOH+Pind:BrdU+/KI67+/DCX- vs. EtOH+Pind:BrdU+/KI67-/DCX+ | 14.37 | -5.726 to 34.47 | No | ns | 0.7592 |
| EtOH+Pind:BrdU+/KI67+/DCX+ vs. EtOH+Pind:BrdU+/KI67-/DCX+ | 41.46 | 22.11 to 60.8 | Yes | **** | <0.0001 |
